# SIVA: diagonal integration of spatial multi-omics data via spatially informed variational autoencoders and anchor guidance

**DOI:** 10.1093/bioinformatics/btag247

**Published:** 2026-07-07

**Authors:** Peng Jiang, Sishuo Chen, Xingye Wu, Juan Liu, Tian Tian

**Affiliations:** School of Artificial Intelligence, School of Computer Science, Wuhan University, Wuhan, 430072, China; School of Artificial Intelligence, School of Computer Science, Wuhan University, Wuhan, 430072, China; School of Artificial Intelligence, School of Computer Science, Wuhan University, Wuhan, 430072, China; School of Artificial Intelligence, School of Computer Science, Wuhan University, Wuhan, 430072, China; School of Artificial Intelligence, School of Computer Science, Wuhan University, Wuhan, 430072, China

## Abstract

**Motivation:**

Understanding cellular states and regulatory programs requires integrative analysis of multiple omics layers. Although recent spatial sequencing technologies allow molecular profiling of cells within their tissue context, paired spatial multi-omics assays are still limited by technical complexity and cost. This creates a pressing need for diagonal integration methods that enable joint analysis of unpaired spatial omics datasets.

**Results:**

We propose **SIVA**, a deep generative framework based on **S**patially-**I**nformed **V**ariational **A**utoencoders with **A**nchor Guidance, for diagonal integration of spatial multi-modal data. SIVA employs modality-specific variational autoencoders (VAEs) with a hybrid latent embedding that integrates Gaussian process and standard Gaussian priors, enabling joint modeling of spatially structured variation and dominant underlying data distributions across modalities. To facilitate cross-modal alignment in the absence of one-to-one cell correspondence, SIVA adopts a dual integration strategy combining global distribution alignment via Maximum Mean Discrepancy and local correspondence guidance using mutual nearest neighbor anchors. Extensive experiments across multiple cross-slice integration scenarios demonstrate that SIVA achieves robust and accurate integration of unpaired spatial omics datasets, consistently outperforming existing methods.

**Availability and implementation:**

The source codes are available at https://github.com/PelenJiang/SIVA

## 1 Introduction

Different omics layers capture distinct aspects of gene regulatory programs and cellular states. A comprehensive understanding of multicellular systems therefore requires integrative analysis of cellular heterogeneity across multiple molecular layers, including gene expression, chromatin accessibility, and protein abundance (Vandereyken *et al*. 2023). Although single-cell sequencing technologies have provided unprecedented insights into these modalities individually, they inherently lack spatial context, which is critical for interpreting cell states within their native microenvironment and tissue architecture.

Recent advances in spatial omics technologies have addressed this limitation by enabling molecular profiling while preserving tissue architecture. However, achieving a holistic view of tissue function often requires the simultaneous interrogation of multiple molecular layers within the same spatial context—a task that remains technically challenging. While emerging spatial multi-omics assays are promising, they are frequently constrained by limited throughput, high experimental cost, or restricted genomic coverage. As a result, a practical and widely adopted strategy is diagonal integration, in which different omics modalities (e.g. transcriptomics and chromatin accessibility) are profiled on separate but related tissue sections and subsequently integrated computationally to recover a coherent multi-omics representation.

Unpaired diagonal integration introduces a distinct set of computational challenges that differentiate it from vertical integration (multiple modalities measured in the same cells) and horizontal integration (the same modality across multiple batches) (Fu *et al*. 2025). First, data generated from distinct tissue sections lack one-to-one correspondence between cells, precluding direct cell-level matching. Second, even adjacent tissue sections may exhibit spatial distortions and biological variability, complicating cross-section alignment. Third, different omics modalities reside in heterogeneous feature spaces with fundamentally different statistical properties, such as the binary nature of chromatin accessibility data versus the overdispersed count distributions of RNA sequencing data. Most existing integration methods were originally developed for dissociated single-cell data, including Seurat canonical correlation analysis (CCA) (Stuart *et al*. 2019), Harmony (Korsunsky *et al*. 2019), scGALA (Jiang *et al*. 2025), scglue (Cao and Gao, 2022), sccross (Yang *et al*. 2024), and scMODAL (Wang *et al*. 2025). Therefore, they often do not explicitly model the spatial dependencies inherent in tissue samples, which limits their effectiveness in diagonal spatial multi-omics integration. More recently, several methods have been proposed for the integration of spatial multi-omics data, including SpatialGlue (Long *et al*. 2024) and COSMOS (Zhou *et al*. 2025). However, these methods are primarily designed for paired spatial multi-omics data, and are, therefore, not directly applicable to unpaired diagonal integration scenarios. To the best of our knowledge, the only existing method designed specifically for spatial diagonal integration tasks is SWITCH (Li *et al*. 2025). However, SWITCH jointly employs two graph neural networks and a generative adversarial network, which can lead to unstable training performance in some cases.

To address these challenges, we propose SIVA (**S**patially **I**nformed **V**ariational Autoencoder with **A**nchor Guidance), a novel computational framework for the diagonal integration of unpaired spatial multi-omics data. SIVA is built upon a spatially informed variational autoencoder (VAE) architecture that explicitly models spatial dependencies in the latent space while accommodating both spatially correlated and independent sources of variation. To enable robust integration across unpaired tissue sections, SIVA adopts a dual alignment strategy, combining global distribution alignment via Maximum Mean Discrepancy (MMD) with local correspondence guidance using Mutual Nearest Neighbors (MNNs). Across multiple datasets, we demonstrate that SIVA consistently outperforms existing methods in integrating spatial omics data. Comprehensive evaluations further validate SIVA’s effectiveness in resolving complex cross-modal variations in unpaired spatial contexts.

## 2 Materials and methods

### 2.1 Overview of SIVA

**Figure 1:**
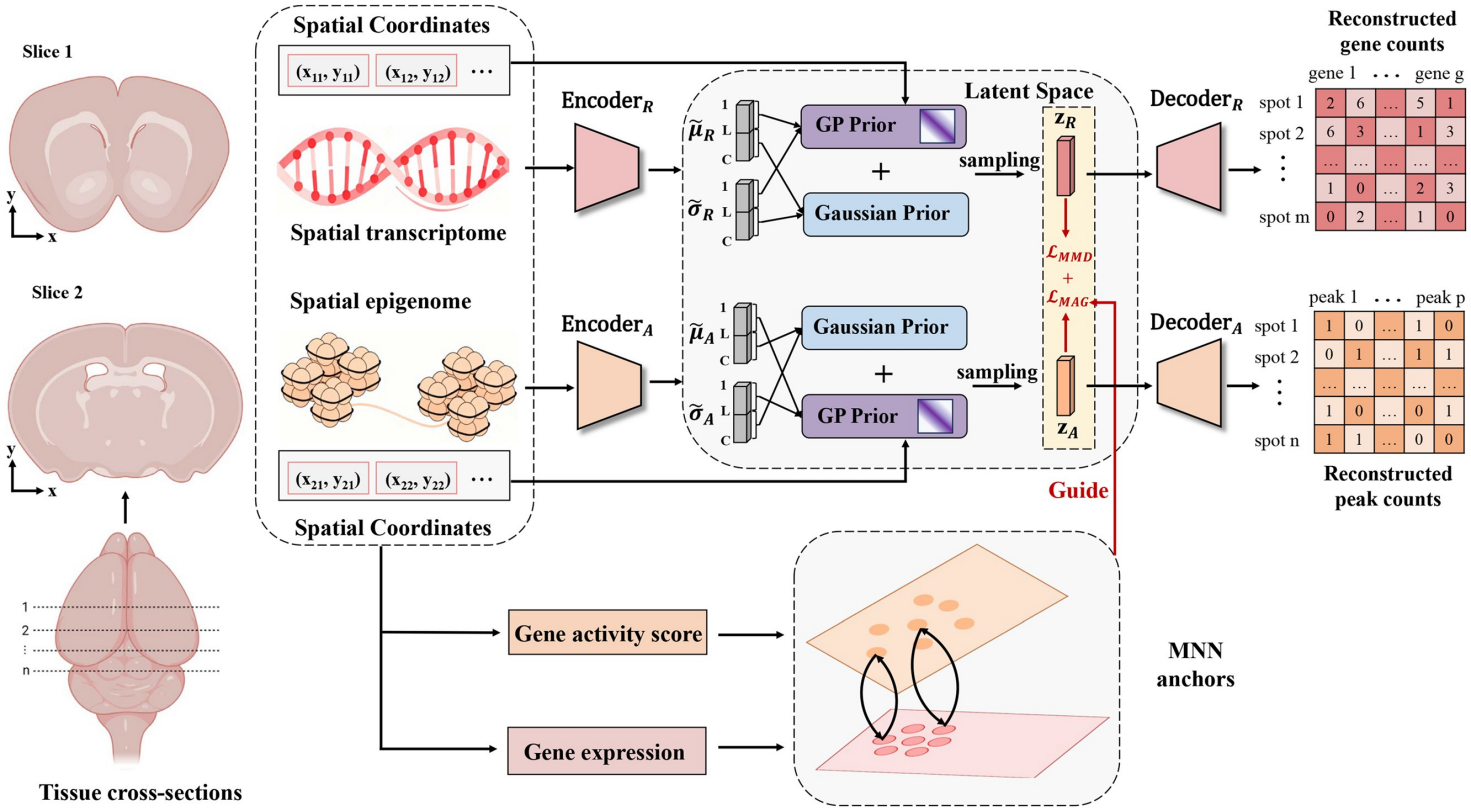
The overall architecture of SIVA. SIVA employs omics-specific VAEs to capture spatially informed latent embeddings using a hybrid latent prior (GP prior for spatially correlated signals and standard Gaussian prior for spatially independent variability). Cross-modal alignment is achieved through a dual alignment strategy, where global alignment is achieved through MMD loss and local alignment is achieved through MNN-based anchor loss. Figure created with Biorender (Agreement number: BI29QFY29D).

SIVA is an unsupervised deep generative framework for the diagonal integration of spatial multi-omics data. It is designed to jointly model multiple spatially resolved molecular modalities by embedding them into a shared latent space while explicitly accounting for spatial dependencies (Fig. 1).

Given spatially resolved omics data from tissue sections, SIVA employs a set of modality-specific VAEs, in which each VAE models one molecular layer. In most applications, two modalities are considered: spatial transcriptomics and spatial chromatin accessibility. Each VAE encodes the input omics data together with their spatial coordinates into a low-dimensional latent space, while the decoder reconstructs the original omics measurements from this latent representation. To account for the fact that not all sources of variation exhibit spatial correlation, SIVA adopts hybrid latent priors that integrate a Gaussian Process (GP) prior for spatially correlated signals with a standard Gaussian prior to model spatially independent variability. Cross-modal integration is achieved through a dual alignment strategy. At the global level, SIVA applies a MMD loss to align the latent distributions learned from different modalities. At the local level, MNNs are identified between modalities and used as anchors to guide fine-scale correspondence between cells. The model is trained by jointly optimizing the evidence lower bound (ELBO) for each VAE together with the MMD alignment loss and the MNN-based anchor loss. This unified objective enables SIVA to learn a shared, spatially informed latent representation that integrates multiple spatial omics modalities.

### 2.2 Spatially informed VAE framework

First, we define the input data. SIVA is designed to process omics data from two distinct modalities, accompanied by corresponding spatial location information. Let $X_{R}$ denotes the spatial transcriptome gene expression counts, containing $N_{R}$ spots and *g* genes. $X_{A}$ denotes the spatial epigenome open chromatin peak counts, containing $N_{A}$ spots and *p* peaks. Each spot in both modalities is associated with spatial coordinates, represented as $Y_{R}\in R^{N_{R}\times 2}$ for spatial transcriptomics and $Y_{A}\in R^{N_{A}\times 2}$ for spatial epigenomics, respectively. The objective of SIVA is to learn a shared latent representation that captures the underlying biological signals across both modalities while accounting for their spatial context. For clarity, the following description employs a unified VAE framework and omits modality-specific labels.

We model the observed data from different omics layers as generated by a low-dimensional latent variable $z\in R^{L}$:


(1) $$\begin{array}{c} p\left(x|y\right)=\int p\left(x|z\right)p(z|y)dz. \end{array}$$

where x represents the observed omics data (gene expression or peak counts) and y denotes the spatial coordinates. The generative process consists of two components: the likelihood p(x|z), which models the generation of observed data given the latent variable, and the prior p(z|y), which captures the spatial dependencies in the latent space conditioned on spatial coordinates. We can derive the ELBO by introducing a variational posterior q(z|x,y):


(2) $$\begin{array}{c} \;\text{log}\;p\left(x|y\right)=\text{log}\;\int p\left(x|z\right)p(z|y)dz\\ =\underset{\;\text{Reconstruction}\;\text{term}\;}{\underset{︸}{E_{q\left(z|x,y\right)}\left[\text{log}\;p\left(x|z\right)\right]}}-\beta \underset{\text{Spatial}\;\text{regularization}\;\text{term}\;}{\underset{︸}{\mathrm{KL}\left(q\left(z|x,y\right)∥p(z|y)\right)}} \end{array}$$

where β controls the weight of the Kullback-Leibler (KL) divergence.

To derive a spatial-dependency-aware latent representation, we introduce a hybrid prior specification: the first *L* dimensions of the latent embedding z are endowed with a GP prior, while the remaining C−L dimensions follow a standard Gaussian prior. Consequently, the KL divergence can be decomposed into a GP term and a standard Gaussian term, which yields the following ELBO formulation:


(3) $$\begin{array}{cc} \text{ELBO}=E_{q\left(z|x,y\right)}\left[\text{log}\;p\left(x|z\right)\right]-\beta [\mathrm{KL}\left(q\left(z^{1:L}|x,y\right)∥p(z^{1:L}|y)\right) & \\ -\mathrm{KL}\left(q\left(z^{L+1:C}|x\right)∥p(z^{L+1:C})\right)], & \end{array}$$

where, $p(z^{1:L}|y)$ follows a GP distribution, and $p(z^{L+1:C})$ follows a standard Gaussian distribution:


$$\begin{array}{c} p(z^{1:L}|y)=\mathrm{GP}(0,K_{NN}),\;\text{and}\;p(z^{L+1:C})=N(0,I), \end{array}$$

where $K_{NN}=k_{\tau }(y,y)$ is the covariance matrix computed from spatial coordinates y using a kernel function $k_{\tau }$. We use the Cauchy kernel in SIVA which can be defined as:


(4) $$k_{\tau }\left(y_{i},y_{j}\right)=\frac{1}{1+{\left\|y_{i}-y_{j}\right\|}^{2}/\tau }.$$

The parameter τ is dynamically trained to model the spatial dependency in the data, and $||y_{i}-y_{j}|{|}^{2}$ represents the Euclidean distance between points.

In the VAE framework, the latent mean $\tilde{\mu }$ and latent variance $\tilde{\sigma }$ are estimated by the encoder network:


$$\tilde{\mu }=f_{\mu }(f(x)),\;\text{and}\;{\tilde{\sigma }}^{2}=\text{exp}(f_{\sigma }(f(x)),$$

where *f*, $f_{\mu }$, and $f_{\sigma }$ are fully connected (FC) layers. exp(·) represents exponential activation function, and the latent posterior distribution is $\tilde{q}(\tilde{z}|x)=N(\tilde{\mu },\sigma^{2})$.

For the last C−L dimensions of standard Gaussian embeddings, the posterior is $q(z^{L+1:C}|x)=\tilde{q}({\tilde{z}}^{L+1:C}|x)=N(\tilde{\mu },{\tilde{\sigma }}^{2})$, thus, we can write the $\mathrm{KL}\left(q\left(z^{L+1:C}|x\right)∥p(z^{L+1:C})\right)$ as:


(5) $$\begin{array}{c} \mathrm{KL}\left(q\left(z^{L+1:C}|x\right)∥p(z^{L+1:C})\right)=\mathrm{KL}\left(q\left(z^{L+1:C}|x\right)∥N(0,I)\right)\\ =\mathrm{KL}\left(N({\tilde{\mu }}^{L+1:C},{\tilde{\sigma }}^{L+1:C^{2}})∥N(0,I)\right)=\frac{1}{2}\sum_{l=L+1}^{C}\left[{\tilde{\mu }}^{l^{2}}+{\tilde{\sigma }}^{l^{2}}-\text{log}\;{\tilde{\sigma }}^{l^{2}}-1\right]. \end{array}$$

For the first *L* dimensions of GP embedding, the posterior is the GP regression of the encoded latent distribution: $q\left(z^{1:L}|x,y\right)=\mathrm{GP}(\tilde{q}\left({\tilde{z}}^{1:L}|x,y)\right)$. Following Pearce (2020), we can rewrite the this posterior distribution as


(6) $$\begin{array}{c} q\left(z^{1:L}|x,y\right)=\frac{\tilde{q}\left({\tilde{z}}_{i}^{1:L}|x_{i}\right)p\left(z^{1:L}|y\right)}{Z^{1:L}(x_{1:N},y_{1:N})}\\ =\frac{1}{Z^{1:L}\left(x_{1:N},y_{1:N}\right)}\prod_{i=1}^{N}\tilde{q}\left({\tilde{z}}_{i}^{1:L}|x_{i}\right)p\left(z^{1:L}|{\tilde{z}}^{1:L},y\right)\\ =\frac{1}{Z^{1:L}\left(x_{1:N},y_{1:N}\right)}\prod_{i=1}^{N}\left\{\prod_{l=1}^{L}N\left({\tilde{z}}_{i}^{l}|{\tilde{\mu }}_{i}^{l},{\tilde{\sigma }}_{i}^{l^{2}}\right)\right\}GP\left(z_{1:N}^{1:L}|{\tilde{z}}_{1:N}^{1:L},y\right), \end{array}$$

where, *N* and *L* denote the number of spots and the number of GP latent dimensions, respectively. $Z^{1:L}\left(x_{1:N},y_{1:N}\right)$ is the sum of the GP marginal likelihoods across all spots. Using this equation, the KL loss of GP embedding can be reformulated:


(7) $$\begin{array}{cc} \mathrm{KL}\left(q\left(z^{1:L}|x,y\right)∥p(z^{1:L}|y)\right)=\sum_{i=1}^{N}E_{q\left(z^{1:L}|x_{i},y_{i}\right)}\left[\text{log}\;\tilde{q}\left({\tilde{z}}^{1:L}|x_{i}\right)\right] & \\ -\sum_{l=1}^{L}\;\text{log}\;Z^{l}\left(x_{1:N},y_{1:N}\right). & \end{array}$$

Following the work of Tian *et al*. (2024), we accelerate computation and enable mini-batch training by incorporating a latent sparse GP regression technique, which utilizes inducing points for scalable inference (Jazbec *et al*. 2021). This enables stochastic optimization and makes the method applicable to large datasets by avoiding the cubic computational complexity of exact GP inference. Finally, the posterior of the GP latent embedding for a mini-batch *b* can be approximated in a closed form as a multivariate Gaussian distribution: $q\left(z^{1:L}|x,y\right)=N(m,B)$, with m and B representing the spatially smoothed mean and covariance matrix, respectively. The resulting KL divergence objective on a mini-batch *b* of data is given by


(8) $$\begin{array}{cc} \mathrm{KL}\left(q\left(z^{1:L}|x,y\right)∥p(z^{1:L}|y)\right) & \\ =-\left[\text{CE}\left(N(m,B)∥N\left({\tilde{\mu }}^{1:L},{\tilde{\sigma }}^{1:L^{2}}\right)\right)+\frac{b}{N}L_{H}\right], & \end{array}$$

where $L_{H}$ is the stochastic estimate of ELBO of GP regression proposed by Hensman *et al*. (2013), and CE is the cross-entropy between the two distributions $q\left(z^{1:L}|x,y\right)$ and $\tilde{q}\left({\tilde{z}}^{1:L}|x,y\right)$. The detailed derivation of Equations (7) and (8) can be referred to Tian *et al*. (2024).

### 2.3 Data likelihoods

#### 2.3.1 Gene likelihood

For mRNA count data, we leverage the negative binomial (NB) distribution to model the gene likelihood. Previous studies illustrated that the NB distribution can effectively characterize the noisy and over-dispersed mRNA count data (Lopez *et al*. 2018, Tian *et al*. 2019). Specifically, the formula is as follows:


(9) $$\begin{array}{c} p(x_{g}^{R}|z)=\prod_{i=1}^{N}\text{NB}(x_{ig}^{R}|\nu_{ig},\theta_{g})\\ =\prod_{i=1}^{N}\frac{\Gamma (x_{ig}^{R}+\theta_{g})}{\Gamma (\theta_{g})\Gamma (x_{ig}^{R}+1)}{\left(\frac{\theta_{g}}{\theta_{g}+\nu_{ig}}\right)}^{\theta_{g}}{\left(\frac{\nu_{ig}}{\theta_{g}+\nu_{ig}}\right)}^{x_{ig}^{R}}, \end{array}$$

where $x_{ig}^{R}$ represents the observed expression count of gene *g* at spot *i*, $\nu_{ig}$ is the predicted mean expression, and $\theta_{g}$ is a trainable dispersion parameter for gene *g*. In our model, the predicted mean expression $\nu_{i}$ is parameterized by decoder networks with respect to the latent embedding $z_{i}$ and the spot-specific library size factor $s_{i}$:


(10) $$\nu_{i}=\mathrm{diag}\left(s_{i}\right)\times \text{exp}(g_{R}(z_{i})),$$

where $g_{R}$ is the decoder network of RNA modality and an exponential activation function is appended to the network, given that the mean is always positive. The library size factor $s_{i}$ accounts for variations in sequencing depth across different spots and is computed as the total counts of all genes at spot *i* divided by the median of total counts across all spots. The reconstruction loss of spot *i*’s gene expression observations is then given by the negative log-likelihood of the NB distribution:


(11) $$\begin{array}{c} L_{{\text{recon}}_{R}}=-\text{log}\;\left(NB\left(x_{i}^{R}|\nu_{i}^{R},\theta^{R}\right)\right). \end{array}$$

#### 2.3.2 Peak likelihood

Spatial ATAC-seq profiles are frequently represented as a binary spot-by-peak matrix, indicating the presence or absence of chromatin accessibility at specific genomic regions. To model the peak likelihood, we use a Bernoulli distribution. Specifically, the observation for peak *q* at spot *i*, denoted $x_{i}^{A}$, follows $\text{Bernoulli}(\lambda_{i}\cdot s_{i}\cdot r_{q})$. Adopting the setting strategy of PeakVI (Ashuach *et al*. 2022), the probability involves a decoder-parameterized term $\lambda_{i}$, alongside spot-specific factor $s_{i}$, and peak-specific factor $r_{q}$ (also estimated by neural networks). The first term is parametrized as:


(12) $$\lambda_{i}=\text{sigmoid}(g_{A}(z_{i})),$$

where z is the latent embedding, and $g_{A}$ is the decoder network of ATAC modality. A sigmoid activation function is applied to constrain the probabilities to the [0,1] interval. The reconstruction loss of spot *i*’s peak observations is


(13) $$L_{{\text{recon}}_{A}}=\text{BCE}\left(\lambda_{i}⊙r\times s_{i},x_{i}^{A}\right),$$

where ⊙ denotes element-wise product, and BCE(·) represents the binary cross-entropy loss.

### 2.4 Dual alignment between modalities

#### 2.4.1 MMD penalty for coarse distribution alignment

To align the distributions of different modalities in the latent space, we utilize the MMD penalty. MMD is a non-parametric metric that measures the distance between two probability distributions based on their samples. Given two sets of samples ${\left\{z_{i}^{R}\right\}}_{i=1}^{N_{R}}$ and ${\left\{z_{j}^{A}\right\}}_{j=1}^{N_{A}}$ from two different modalities, the MMD loss is defined as:


(14) $$\begin{array}{cc} L_{\text{MMD}}=\frac{1}{N_{R}(N_{R}-1)}\sum_{i\neq j}k\left(z_{i}^{R},z_{j}^{R}\right) & \\ +\frac{1}{N_{A}(N_{A}-1)}\sum_{i\neq j}k\left(z_{i}^{A},z_{j}^{A}\right)-\frac{2}{N_{R}N_{A}}\sum_{i,j}k\left(z_{i}^{R},z_{j}^{A}\right) & \end{array}$$

Here, *k* is a kernel function that measures the similarity between two latent embeddings. We use the Inverse Multi-Quadratic kernel, which is defined as:


(15) $$k\left(x,y\right)=\frac{Q}{Q+{\left\|x-y\right\|}^{2}},$$

where *Q* is a constant that controls the width of the kernel.

#### 2.4.2 MNN anchor for fine-grained integration guidance

To achieve robust cross-modal integration, it is essential to exploit correspondence between modalities. To this end, we employ the MNN approach to identify cross-modal anchors based on shared features. Specifically, we utilize the FindIntegrationAnchors function from the Seurat package to implement the identification of MNN pairs. The process is as follows: we first compute the gene expression profiles for both modalities. For spatial ATAC-seq data, we calculate gene activity scores using the established method (Stuart *et al*. 2021). Genes shared between the two modalities are then projected into a common low-dimensional space using CCA. Next, we identify MNN pairs between the two modalities in the shared latent space. These pairs serve as anchors to guide the fine-scale alignment of the latent representations. The MNN anchor guidance (MAG) loss is defined as the mean squared error between the latent embeddings of the MNN pairs:


(16) $$L_{\text{MAG}}=\frac{1}{\left|G\right|}\sum_{(i,j)\in G}||z_{i}^{R}-z_{j}^{A}|{|}^{2},$$

where G denotes the set of MNN anchor pairs, and ||·|| represents the Euclidean distance.

### 2.5 Overall training objectives

The overall training objective of SIVA consists of: the reconstruction losses for gene expression and peak counts, the KL divergence for latent regularization, the MMD penalty for coarse distribution alignment, and the MAG loss for fine-grained integration. The total loss function $L_{\text{Total}}$ is given by:


(17) $$L_{\text{total}}=\sum_{i\in \{R,A\}}\left(L_{{\text{recon}}_{i}}+\beta_{i}L_{{\text{KL}}_{i}}\right)+\alpha L_{\text{MMD}}+\gamma L_{\text{MAG}},$$

where β,γ,α are weighting factors. We use the dynamic VAE technique (Shao *et al*. 2022) to automatically tune the weight of the KL loss β. The α and γ are set to balance the contributions of MMD loss and MAG loss.

## 3 Results

### 3.1 Datasets and annotations

We utilized six samples from the MISAR-seq dataset (Jiang *et al*. 2023), two samples from the Spatial ATAC-RNA-seq dataset (Zhang *et al*. 2023), the mouse embryonic development E15-rep1 sample from the Spatial ATAC-seq (Llorens-Bobadilla *et al*. 2023), and the Human lymph node dataset from SpatialGLUE (Long *et al*. 2024). For a detailed introduction to the dataset and the corresponding annotations, please refer to the Supplementary Note 1, available as supplementary data at *Bioinformatics* online.

### 3.2 Implementation details

We utilized the SCANPY (Wolf *et al*. 2018) package in Python and Signac (Stuart *et al*. 2021) package in R to preprocess the raw data. For the spatial transcriptomics data, we normalized the raw gene expression counts using total-count normalization and calculated the library size factor $s_{i}=\frac{s_{i}}{\text{median}\left({\left\{s_{j}\right\}}_{j=1,\ldots ,N}\right)}$, for each spot. Then, 2000 highly variable genes were selected using the Seurat “vst” method and the raw data was log-transformed and scaled. The preprocessed expression matrix was used as the input for our SIVA model, and we used the original count matrix when calculating the NB loss of genes. For the spatial chromatin accessibility data, we used the CallPeaks function from Signac, which utilized MACS2 (Zhang *et al*. 2008) to call peaks. For benchmarking purposes, spatial chromatin accessibility data were additionally converted to gene-level activity scores using Signac, as several comparison methods (e.g. Harmony and Seurat CCA) require gene-level inputs. This conversion was not required for SIVA, which directly models peak-level chromatin accessibility data. Next, binarization of the peak count was performed, wherein any value exceeding 0 was assigned a value of 1. We used peaks overlapping with the gene bodies or proximal promoter regions of highly variable genes as input for SIVA model training.

SIVA was implemented using the PyTorch (Paszke *et al*. 2019) library. We used the following architecture for SIVA: The default layer sizes of RNA encoder are set to (128, 64) and (64, 128) for the decoder. For the VAE used for ATAC modality, default layer sizes of (1024, 128) were set for the encoder, and (128, 1024) for the decoder. The latent embedding dimension was set to 20 (4 for GP and 16 for normal Gaussian) and all hidden layers were activated by ELU (exponential linear unit) function and the batch normalization technique. The remaining training details are provided in the Supplementary Note 2, available as supplementary data at *Bioinformatics* online.

From systematic benchmarks, we selected the following methods: Seurat CCA (Stuart *et al*. 2019), Harmony (Korsunsky *et al*. 2019), bindSC (Dou *et al*. 2022), LIGER (Welch *et al*. 2019), Online iNMF (Gao *et al*. 2021), scGALA (Jiang *et al*. 2025), scglue (Cao and Gao 2022), sccross (Yang *et al*. 2024), monae (Tang *et al*. 2024), scMODAL (Wang *et al*. 2025), and SWITCH (Li *et al*. 2025). All competing methods were applied with their respective recommended default hyperparameter settings and preprocessing steps. Each method was executed with three random seeds. For the systematic scalability test, all methods were run on a Linux workstation with 96 CPU cores [Intel(R) Xeon(R) Gold 6267C CPU @ 2.60 GHz], 1510 GB of RAM and an 80 GB NVIDIA A100 GPU. The comparison of running time and GPU memory usage is listed in Table 1, available as supplementary data at *Bioinformatics* online.

**Table 1:** Ablation study results for components in SIVA.

Methods	Mean bio score	Mean omics score
w/o $L_{\text{MAG}}$	0.5772 ± 0.0207	0.9429 ± 0.0044
w/o $L_{\text{MMD}}$	0.5044 ± 0.0376	0.5017 ± 0.0611
w/o GP priors	0.4910 ± 0.0106	0.9576 ± 0.0036
SIVA	0.6246 ± 0.0155	0.9632 ± 0.0077

### 3.3 Evaluation metrics

To evaluate the performance of SIVA, we adopted the following metrics referring to previous studies (Cao and Gao 2022, Luecken *et al*. 2022, Fu *et al*. 2025): for biology conservation, mean average precision (MAP), cell type average silhouette width, normalized mutual information, adjusted rand index (ARI), and Graph local inverse Simpson’s index of cell types (cLISI); for omics mixing, omics ASW (oASW), k-nearest neighbor batch effect test, graph connectivity, Seurat alignment score, and LISI of omics (iLISI). Besides, the FOSCTTM was used to evaluate the single-spot level alignment accuracy, which was computed on two datasets with known spot-to-spot pairings. The detailed definitions of these metrics are provided in Supplementary Note 3, available as supplementary data at *Bioinformatics* online. For the ablation study, we used the mean values of five biology conservation metrics and the mean values of five omics mixing metrics as the final evaluation metrics. For the purpose of benchmarking, we calculated the overall score, $S_{\text{overall}}$, which was calculated by taking the weighted mean of the biology conservation score, $S_{\text{bio}}$, and the omics mixing score, $S_{\text{omics}}$:


$$\begin{array}{cc} S_{\text{overall}} & =0.6\times S_{\text{bio}}+0.4\times S_{\text{omics}},\\ S_{\text{bio}} & =\frac{1}{\left|M_{\text{bio}}\right|}\sum_{m_{i}\in M_{\text{bio}}}s\text{cale}\left(m_{i}\right),\\ S_{\text{omics}} & =\frac{1}{\left|M_{\text{omics}}\right|}\sum_{m_{i}\in M_{\text{omics}}}s\text{cale}\left(m_{i}\right). \end{array}$$

Here, $M_{\text{bio}}$ and $M_{\text{omics}}$ are the sets of metrics used for biology conservation and omics mixing, and scale(·) is the min-max scaling function that maps the metric values to the range of [0,1].

### 3.4 Systematic benchmarking for spatial multi-omics integration

**Figure 2:**
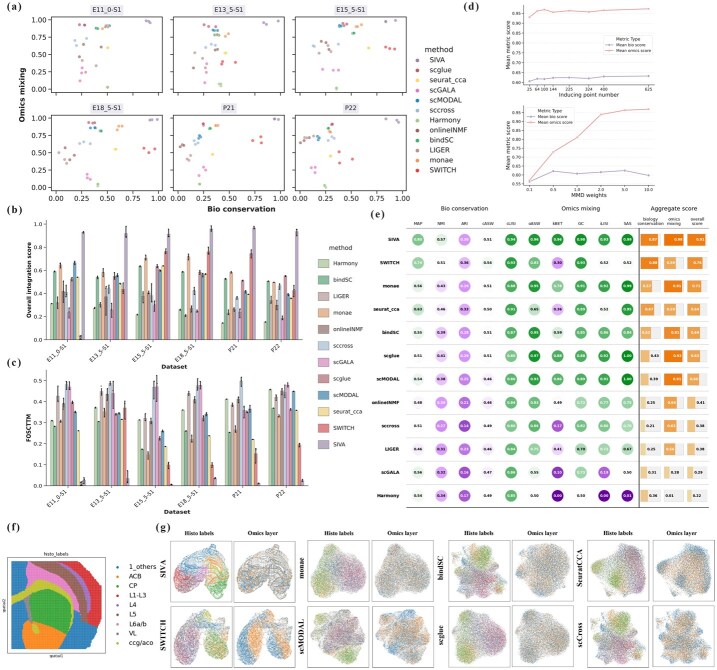
Systematic benchmarks of integration performance. (a) Biological conservation score versus omics mixing score for different integration methods across six datasets. (b) Overall integration score of different methods (*n* = 3 repeats with different model random seeds). (c) Spot-level alignment error (FOSCTTM) of different methods across six datasets. (d) Mean bio score and omics score of different hyperparameter settings: inducing point steps (top) and MMD weights (bottom). (e) All specific metrics of the benchmark results on the E15.5-S1 dataset. (f) Manual annotation of the P22 sample. (g) UMAP visualization of the latent embeddings generated by each method, colored by histological labels (left panel) and omics layers (right panel).

To systematically benchmark SIVA, we conducted a comprehensive comparison against several state-of-the-art methods across multiple paired multi-omics datasets (E11.0-S1, E13.5-S1, E15.5-S1, E18.5-S1, P21, and P22). A robust integration algorithm should harmonize distinct omics layers to align corresponding cell states while retaining intrinsic biological signals. In this regard, SIVA demonstrates exceptional performance compared to existing methods. It consistently achieves the highest overall scores across all benchmark datasets by simultaneously maximizing both biological conservation and omics mixing metrics (Fig. 2a and b). To provide a finer-scale assessment of integration performance, we evaluated spot-level alignment error using the FOSCTTM (fraction of samples closer than true match) metric. On all six datasets, SIVA achieved the lowest FOSCTTM values, with all results below 0.1, significantly outperforming other methods (Fig. 2c).

Using the SIVA model with unified parameters (described in the section of implementation details), we first systematically evaluated SIVA on the E15.5-S1 dataset. Figure 2e illustrates the detailed comparison of specific metrics. Overall, deep learning-based methods (SWITCH, monae, scglue, scMODAL) generally outperformed traditional machine learning-based integration methods (Harmony, LIGER, online iNMF). Notably, Harmony and scGALA exhibited relatively poor performance, with their UMAP results (Fig. 2, available as supplementary data at *Bioinformatics* online) revealing significant batch effects between omics layers, indicating they may be ill-suited for spatial multi-omics data. Specifically, scGALA integrates data by refining anchors initially derived from Seurat CCA to select higher-quality candidates for the final alignment. However, on spatial multi-omics data, it generated an insufficient number of high-quality anchors, ultimately leading to integration failure. In contrast, the original Seurat CCA was able to adapt effectively to the integration of spatial multi-modal data, primarily because it retained a sufficient number of anchors. Among all the methods, SIVA demonstrated superior performance compared to all other benchmarked methods. Specifically, it achieved the highest overall integration score of 0.91, significantly outperforming the second-best method, SWITCH (score = 0.78), which also incorporates spatial information by graph attention networks.

**Figure 3:**
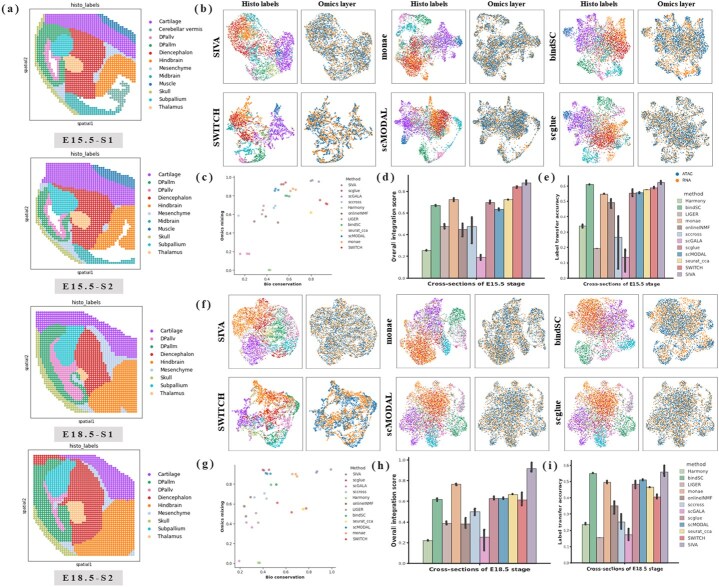
Cross-slice multi-omics integration results of SIVA. (a) Manual annotations based on the anatomical regions of the MISAR-seq E15.5 and E18.5 sections (Jiang *et al*. 2023). (b) UMAP visualization of the latent embeddings generated by SIVA, SWITCH, monae, scMODAL, bindSC, and scglue on E15.5 stage. (c) Biological conservation score versus omics mixing score for integrating cross-sections of E15.5 stage. (d) Overall integration score of different methods on E15.5 stage. (e) Label transfer accuracy of different methods on E15.5 cross-sections. (f) UMAP visualization of the latent embeddings generated by different methods on E18.5 stage. (g) Biological conservation score versus omics mixing score for integrating cross-sections of E18.5 stage. (h) Overall integration score of different methods on E18.5 stage. (i) Label transfer accuracy of different methods on E18.5 cross-sections.

**Figure 4:**
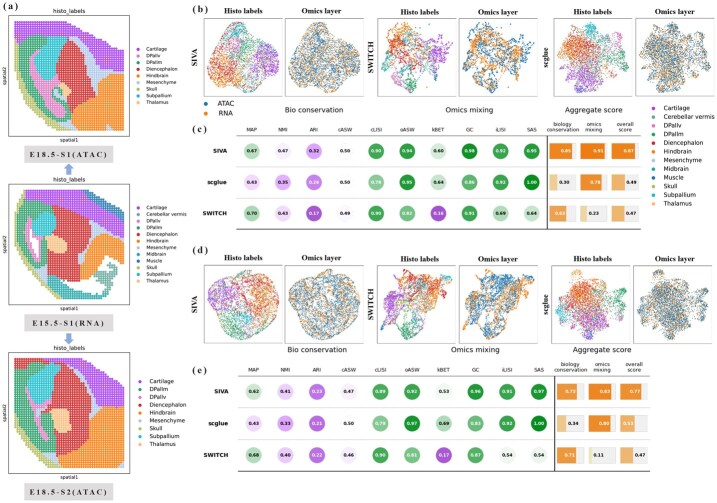
Cross-stage integration results of SIVA. (a) Manual annotation of E15.5-S1, E18.5-S1 and E18.5-S2 samples. (b) UMAP visualization of the latent embeddings generated by SIVA, SWITCH and scglue for integrating E15.5-S1 and E18.5-S1 samples. (c) Detailed evaluation metric scores for integrating E15.5-S1 and E18.5-S1 samples. (d) UMAP visualization of the latent embeddings generated by SIVA, SWITCH and scglue for integrating E15.5-S1 and E18.5-S2 samples. (e) Detailed evaluation metric scores for integrating E15.5-S1 and E18.5-S2 samples.

We further evaluated SIVA on a larger dataset, P22, which contained many more spots than MISAR-seq E15.5-S1 data. The UMAP results (Fig. 2g and Fig. 3, available as supplementary data at *Bioinformatics* online) revealed that SIVA was capable of capturing the complex spatial patterns of chromatin accessibility and gene expression, and accurately integrating them into a common space. SWITCH failed to effectively remove batch effects, resulting in separated clusters for the same cell types across slices; meanwhile, most other competing methods exhibited over-mixing, leading to blurred boundaries between distinct anatomical regions. In contrast, SIVA achieved a balanced integration, harmonizing information across modalities while maintaining clear anatomical structure. Additional comparison of the P22 dataset was provided in Fig. 4, available as supplementary data at *Bioinformatics* online.

#### 3.4.1 Ablation study

To further investigate the contribution of each component in the overall loss function, based on E15.5-S1 sample of MISAR-seq dataset, we performed an ablation study to assess the contribution of each component in the overall loss function. The quantitative results were presented in Table 1. We observed that the incorporation of the GP prior played a critical role in preserving cellular heterogeneity, while the introduction of the MMD loss was significant for facilitating modality mixing.

We further investigated the robustness of the model with respect to the number of inducing points in the GP prior. As illustrated in Fig. 2d, the model’s performance stabilized when the number of inducing points exceed 64. Considering the scale of the datasets, particularly the P22 dataset, which contains 9215 spots, we selected 225 inducing points as the default parameter for our experiments. Regarding the MMD loss weight for modality mixing, we found that the mixing performance tended to stabilize when the weight exceeded 2. However, at a weight of 10, the mean biological conservation score began to decline, indicating potential over-integration. Consequently, we adopted an MMD weight of 5 to balance mixing efficiency with biological conservation.

As for MAG loss weight, we have conducted an ablation study to investigate the selection of MAG loss weight. We extensively examined its influence on the model within the range of 0.01–50. We found that the model’s performance was relatively stable from 0.1 all the way up to around 5. However, starting from 10, the model’s performance began to decline. When the weight exceeded 20, it even performed worse than without the MAG loss. We also conducted ablation experiments on different GP dimensions and different anchor scores. The detailed ablation results are provided in Fig. 5, available as supplementary data at *Bioinformatics* online.

### 3.5 SIVA achieves better cross-slice multi-omics integration

To further validate the robustness of SIVA in integrating spatial datasets, we applied it to cross-sections of mouse embryos at two developmental stages: E15.5 (S1 and S2) and E18.5 (S1 and S2) (Fig. 3a). Specifically, we used the ATAC data from section 1 and the RNA data from section 2 to integrate.

Visual inspection of the low-dimensional embeddings (Fig. 3b and f) revealed that SIVA effectively harmonizes the omics layers from different slices. As shown in the “Omics layer” columns, SIVA achieved thorough mixing of the two sections within each anatomical region, indicating successful removal of slice-specific batch effects. In contrast, other methods such as SWITCH and bindSC often exhibited residual separation between slices or failed to maintain clear boundaries between distinct anatomical structures (as seen in the “Histo labels” columns).

Quantitatively, SIVA consistently outperformed 11 state-of-the-art methods. In the evaluation of integration quality (Fig. 3c and g), SIVA occupied the optimal position in the scatter plots of biological conservation versus omics mixing for both E15.5 and E18.5 datasets, striking the best balance among all tested methods. This is further corroborated by the overall integration scores (Fig. 3d and h), where SIVA achieved the highest rankings.

Furthermore, we assessed the practical utility of the alignment through label transfer. To this end, we trained a simple support vector machine (SVM) classifier on the integrated embeddings of S2, then used S2 labels to predict S1 labels and evaluate prediction accuracy. As shown in Fig. 3e and i, SIVA demonstrated superior performance in transferring anatomical labels between slices. This confirmed that SIVA not only integrated the data statistically but also preserved biologically meaningful structure, enabling accurate downstream cross-slice analysis.

### 3.6 SIVA enables the integration of slices from different stages

To evaluate the capability of SIVA in handling challenging integration scenarios, we applied it to integrate mouse embryo slices from different developmental stages, using RNA data from E15.5 and ATAC data from E18.5. This task is particularly difficult due to substantial anatomical and molecular changes that occur during development, in addition to discrepancies arising from different omics modalities. As shown in Fig. 4, SIVA achieved the highest overall score and successfully harmonized the omics layers while maintaining distinct separation between anatomical regions. In contrast, SWITCH demonstrated respectable performance in biological conservation, but it failed to effectively eliminate batch effects between different modalities. Conversely, although scglue integrated the two modalities well, it showed limitations in preserving biological heterogeneity of anatomical regions.

## 4 Discussion

In this study, we proposed SIVA, a novel deep generative framework designed to address the challenge of diagonal integration in spatial multi-omics data. Unlike existing integration methods, the core advantage of SIVA lies in its carefully designed hybrid latent prior, which combines a GP to explicitly capture spatially correlated biological signals with a standard Gaussian distribution to model spatially independent variability. Furthermore, SIVA employs a coarse-to-fine dual alignment strategy, utilizing MMD for global distribution alignment and MNN anchors for fine-grained local correspondence, thereby establishing robust mapping across different modalities and tissue slices.

Extensive benchmarking on mouse brain datasets covering multiple developmental stages (E11.0 to P22) demonstrated that SIVA achieves the optimal balance between biological conservation and omics mixing, significantly outperforming 11 state-of-the-art methods, including SWITCH, scglue, bindSC, etc. These results highlight the advantage of explicitly modeling spatial dependencies when integrating spatial multi-omics data, particularly in settings characterized by spatial distortion, modality-specific noise, and the absence of one-to-one cell correspondence. By preserving anatomically meaningful structure while enabling effective cross-modal alignment, SIVA provides a robust computational framework for dissecting cellular heterogeneity in complex tissues.

We further expanded the application of SIVA, attempting to integrate spatial RNA and spatial ADT in the human lymph node dataset. Additionally, we constructed real unpaired samples by combining the MISAR-seq E15.5-S1 sample with the Spatial ATAC-seq E15-rep1 sample. We found that the performance of SIVA was still superior to that of Seurat CCA or scglue, as shown in Figs 6 and 7, available as supplementary data at *Bioinformatics* online.

Despite its impressive performance, several challenges and opportunities remain. In this work, SIVA is primarily evaluated on the integration of spatial transcriptomics and spatial chromatin accessibility data. Future extensions could incorporate additional spatial modalities, such as spatial proteomics, spatial metabolomics, and spatial imaging data, further enhancing the comprehensiveness with which tissue organization and cellular states can be characterized. Moreover, extending SIVA to model temporal dynamics or longitudinal spatial data may enable deeper insights into developmental processes and disease progression. Together, these directions point toward a broader role for spatially informed generative models in advancing the integration of spatial multi-omics.

## Supplementary Material

btag247_Supplementary_Data

## Data Availability

The source codes are available at https://github.com/PelenJiang/SIVA. The processed data is freely available at: https://zenodo.org/records/20034790.
