# Supplementary material for: SIVA: diagonal integration of spatial multi-omics data via spatially informed variational autoencoders and anchor guidance
Source: Bioinformatics. 2026 Jul 7;42(Suppl 1):btag247. doi: 10.1093/bioinformatics/btag247 (PMC13340223; doi:10.1093/bioinformatics/btag247)
Supplement: btag247_Supplementary_Data [file btag247_supplementary_data.pdf]

Supplementary Materials:
SIVA: Diagonal Integration of Spatial Multi-omics Data via Spatially-Informed Variational Autoencoders and Anchor Guidance

Peng Jiang, Sishuo Chen, Xingye Wu, Juan Liu, and Tian Tian∗

School of Artificial Intelligence, School of Computer Science, Wuhan University, Wuhan, China

Supplementary Figures

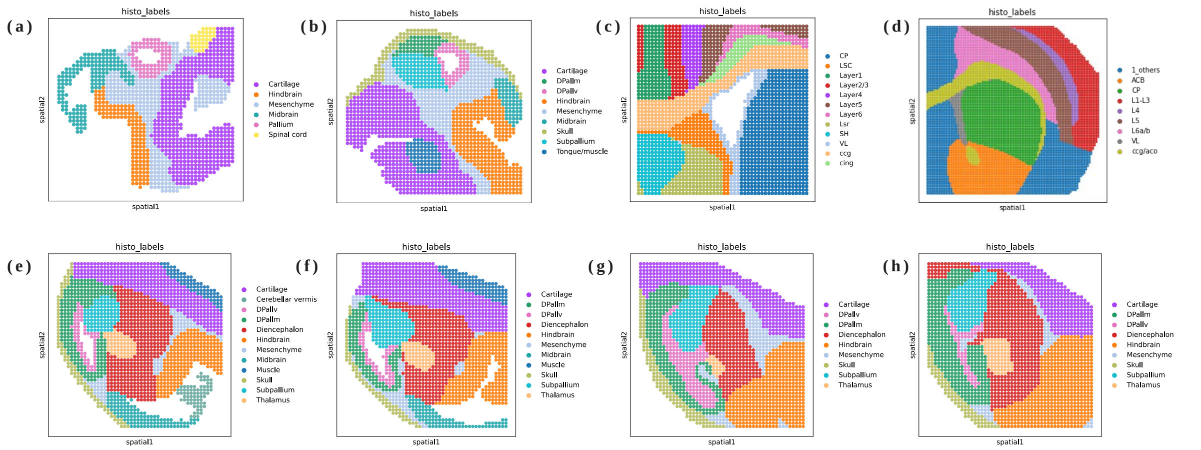

Supplementary Fig. 1. Manual annotation of MISAR-seq and Spatial ATAC-RNA-seq dataset. (a) E11.5-S1 sample. (b) E13.5-S1 sample.(c) P21 sample. (d) P22 sample. (e) E15.5-S1 sample. (f) E15.5-S2 sample. (g) E18.5-S1 sample. (h) E18.5-S2 sample.

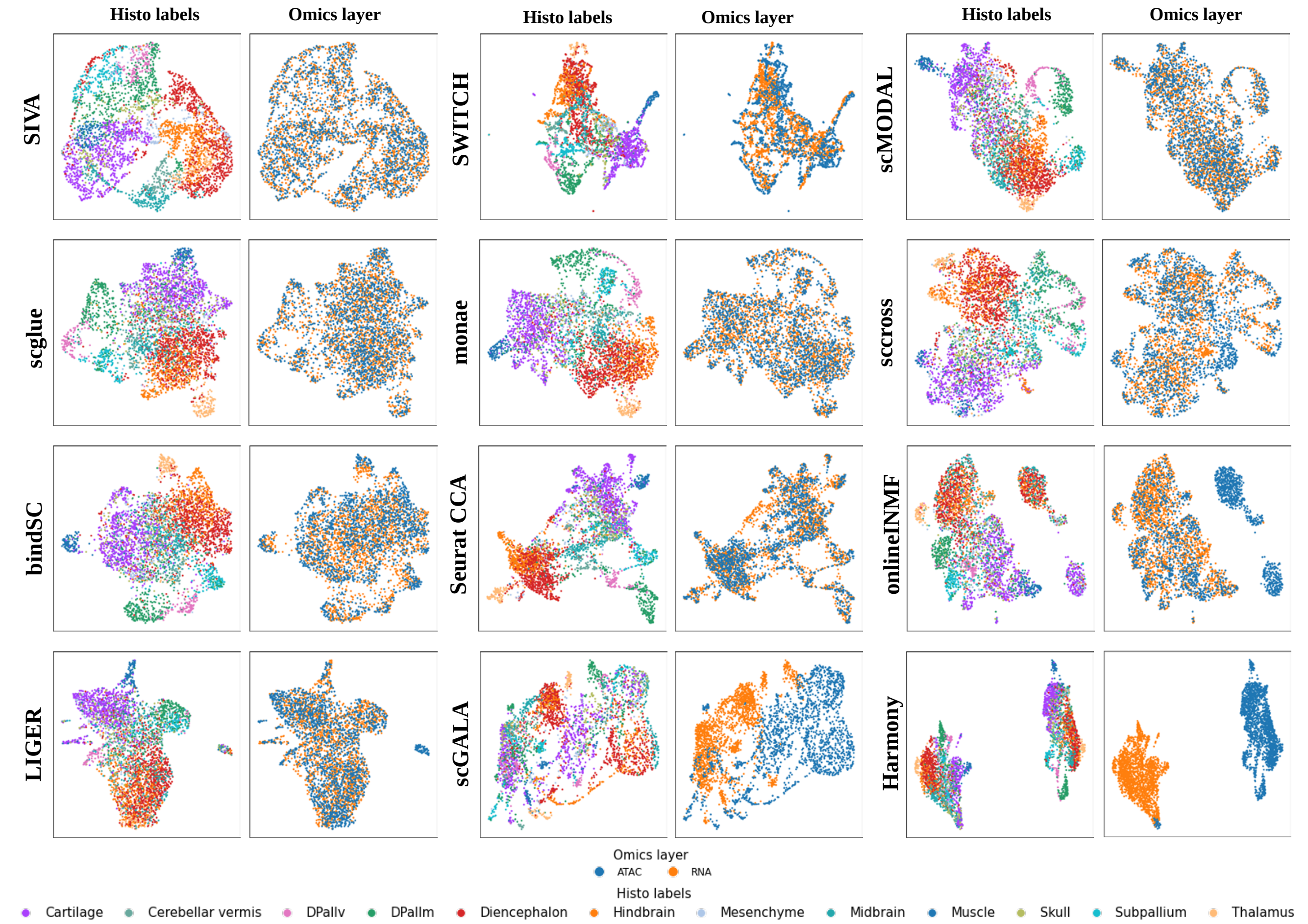

Supplementary Fig. 2. UMAP visualization of the latent embeddings generated by different methods on E15.5-S1 sample, colored by histological labels (left panel) and omics layers (right panel).

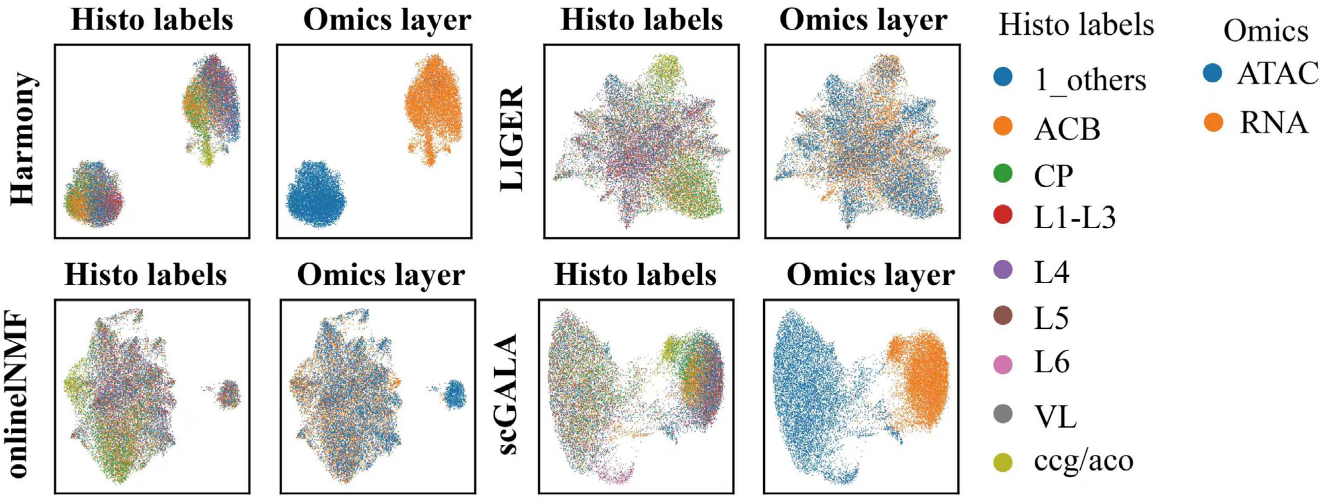

Supplementary Fig. 3. UMAP visualization of the latent embeddings on P22 sample generated by Harmony, online iNMF, LIGER, and scGALA, colored by histological labels (left panel) and omics layers (right panel).

	Bio conservation					Omics mixing					Aggregate score		
	MAP	NMI	ARI	cASW	cLISI	oASW	kBET	GC	iLISI	SAS	biology conservation	omics mixing	overall score
**SIVA**	0.83	0.56	0.35	0.50	0.99	0.96	0.69	0.99	0.86	0.99	0.91	0.97	0.94
**scglue**	0.30	0.22	0.15	0.49	0.66	0.96	0.67	0.87	0.91	1.00	0.31	0.91	0.55
**bindSC**	0.30	0.18	0.14	0.49	0.65	0.97	0.69	0.77	0.90	0.95	0.28	0.85	0.51
**monae**	0.31	0.23	0.15	0.48	0.71	0.91	0.54	0.78	0.91	0.98	0.29	0.80	0.50
**sccross**	0.35	0.19	0.11	0.49	0.69	0.93	0.21	0.87	0.79	0.81	0.31	0.71	0.47
**SWITCH**	0.45	0.34	0.22	0.47	0.83	0.79	0.12	0.86	0.20	0.46	0.43	0.42	0.43
**scMODAL**	0.29	0.11	0.06	0.49	0.63	0.91	0.37	0.81	0.78	0.83	0.18	0.71	0.39
**seurat_cca**	0.31	0.19	0.12	0.49	0.68	0.57	0.15	0.83	0.28	0.98	0.30	0.45	0.36
**LIGER**	0.27	0.10	0.06	0.46	0.62	0.87	0.49	0.77	0.82	0.88	0.08	0.72	0.34
**onlineINMF**	0.24	0.07	0.03	0.47	0.60	0.85	0.44	0.73	0.81	0.86	0.04	0.68	0.30
**scGALA**	0.28	0.10	0.04	0.48	0.63	0.53	0.08	0.81	0.04	0.52	0.13	0.26	0.19
**Harmony**	0.31	0.16	0.09	0.49	0.65	0.48	0.00	0.65	0.00	0.02	0.24	0.02	0.15

Supplementary Fig. 4. All specific metrics of the benchmark results on the P22 dataset.

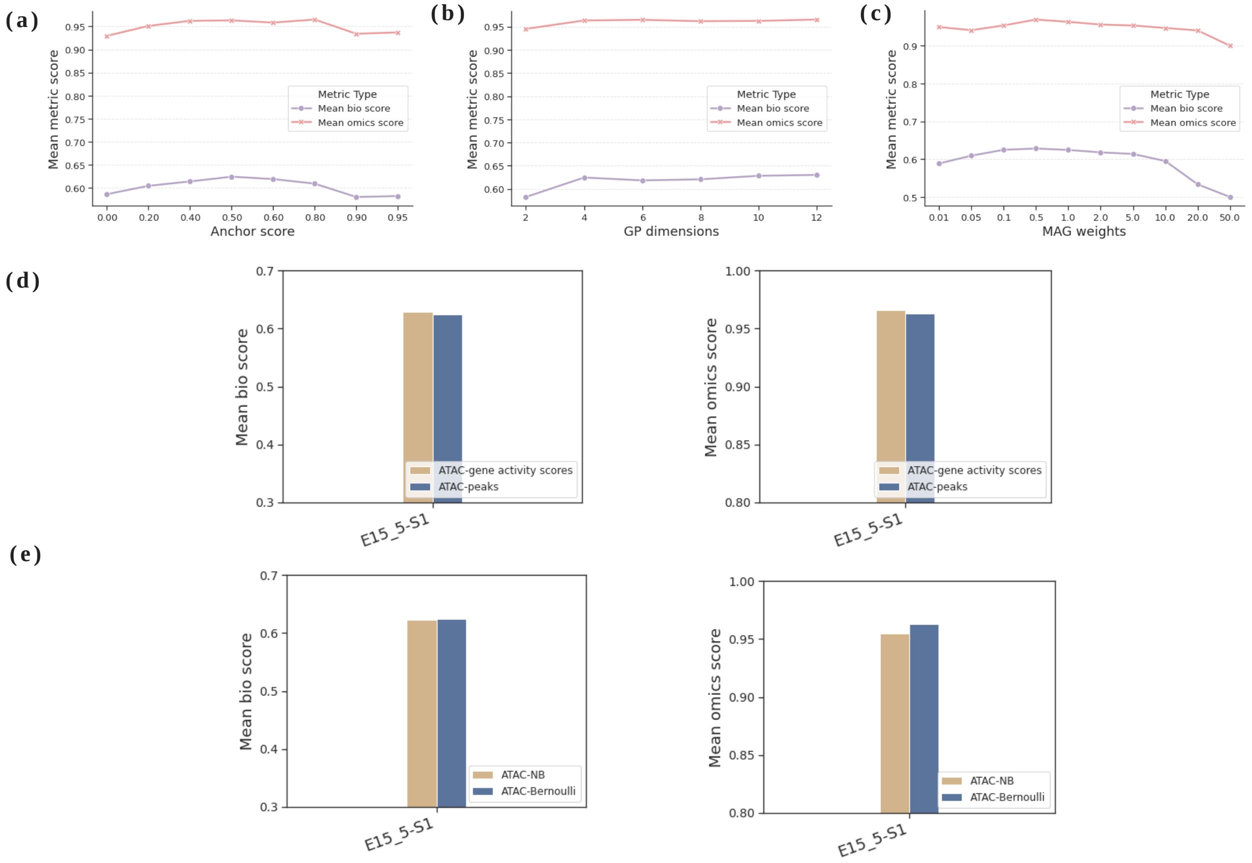

Supplementary Fig. 5. All ablation results on MISAR-seq E15.5-S1 sample.

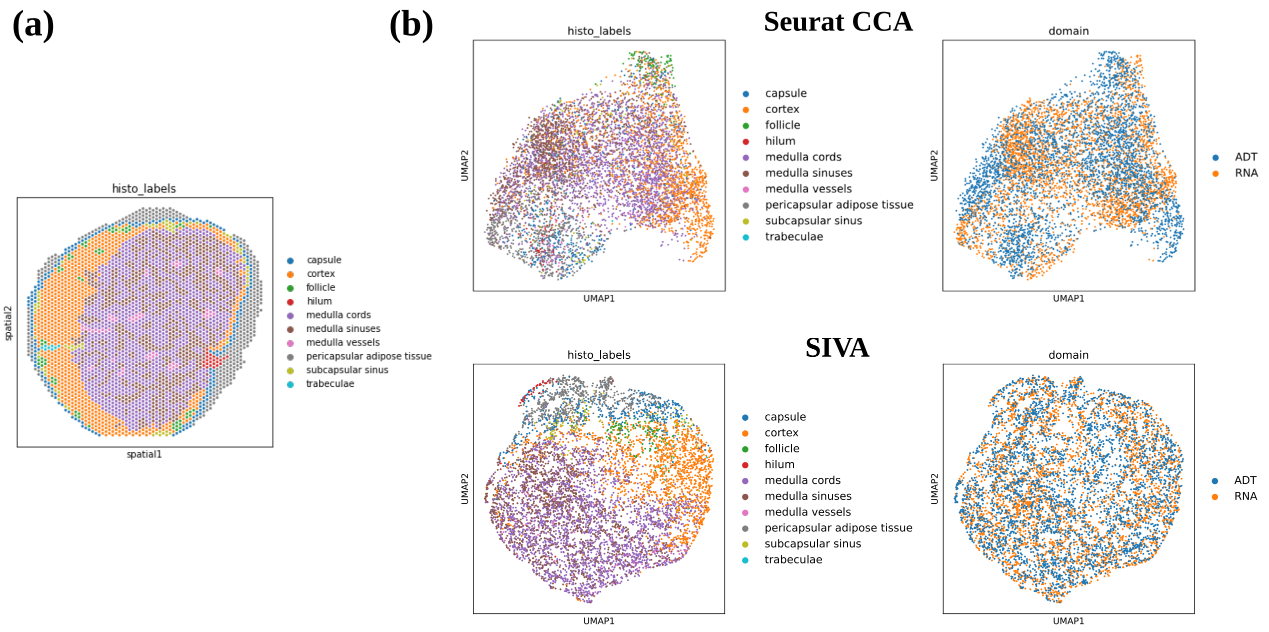

Supplementary Fig. 6. Integration results on Human lymph node dataset. (a) Manual annotation of the dataset. (b) UMAP visualization of the latent embeddings generated by Seurat CCA and SIVA, colored by histological labels (left panel) and omics layers (right panel).

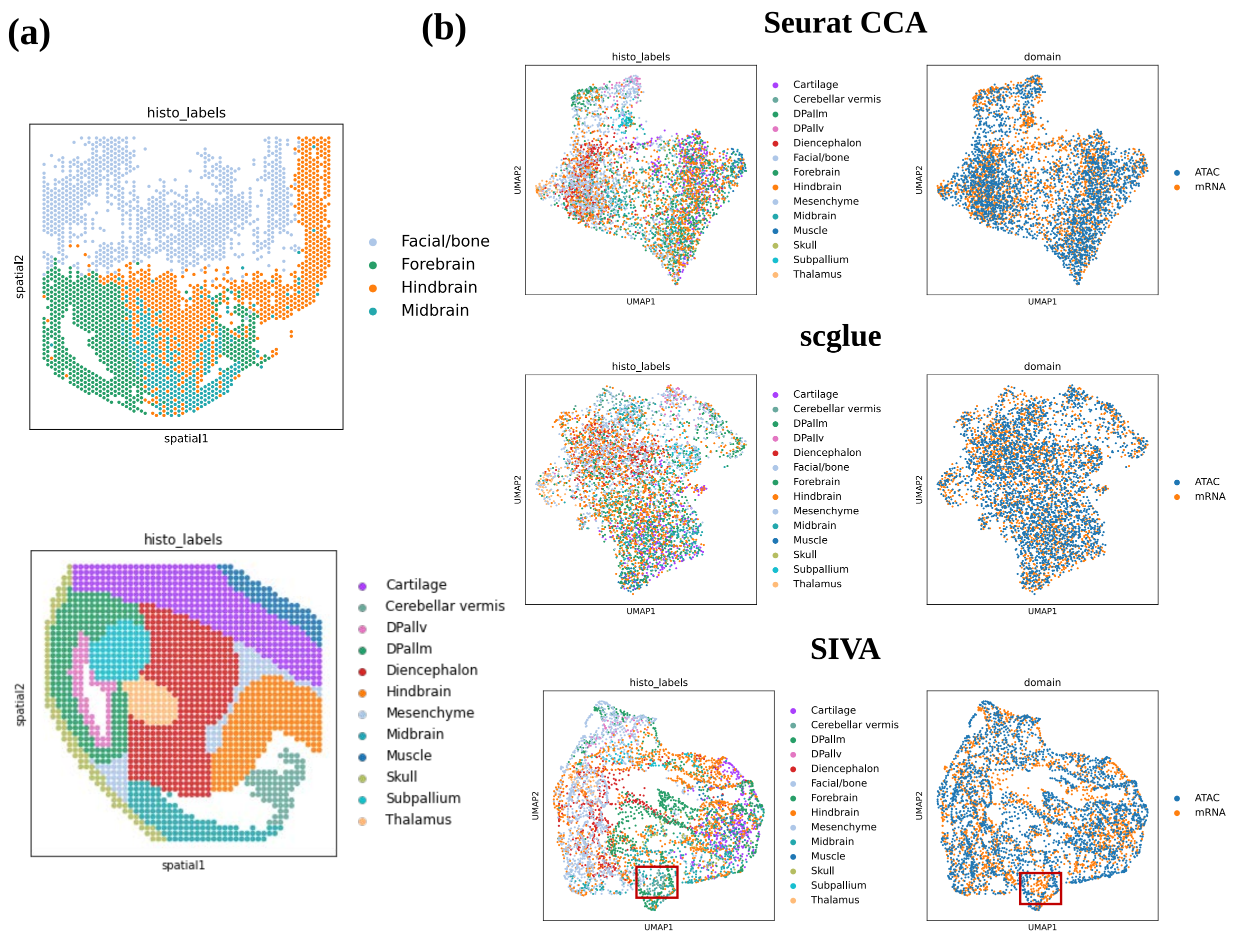

Supplementary Fig. 7. Integration results on MISAR-seq E15.5-S1 RNA and spatial ATAC-seq E15-rep1 dataset. (a) Manual annotation of the two datasets. (b) UMAP visualization of the latent embeddings generated by Seurat CCA, scglue and SIVA, colored by histological labels (left panel) and omics layers (right panel).

Supplementary Tables

Supplementary Table 1. Comparison of runtime and GPU memory usage for various methods.

Method	MISAR-seq E15.5-S1 (1949 spots)			Spatial ATAC-RNA-seq P22 (9215 spots)		
	Batch Size	GPU Memory	Runtime	Batch Size	GPU Memory	Runtime
Harmony	—	—	2 min	—	—	4 min
Seurat CCA	—	—	4 min	—	—	7 min
LIGER	—	—	25 s	—	—	68 s
onlineINMF	—	—	4 s	—	—	12 s
bindSC	—	—	2 min	—	—	4 min
SWITCH	—	3822 MiB	4 min	—	13328 MiB	19 min
scglue	512	2236 MiB	44 min	512	1868 MiB	87 min
sccross	512	17388 MiB	2 h	512	10384 MiB	3 h
scGALA	—	2796 MiB	10 min	—	11832 MiB	81 min
monae	512	2650 MiB	70 min	512	2278 MiB	2 h
scMODAL	512	7034 MiB	96 min	512	7308 MiB	2 h
SIVA	512	7700 MiB	82 min	512	8106 MiB	6 h

Supplementary Note 1: Datasets and annotations

MISAR-seq dataset [4]. The spatially resolved gene expression and chromatin accessibility data of mouse embryonic brain profiled by MISAR-seq platform is available under the National Genomics Data Center accession no. OEP003285 (www.biosino.org/node/project/detail/OEP003285). We analyzed six mouse embryonic brain samples covering developmental stages E11.0 to E18.5, with representation at E11.0 (S1), E13.5 (S1), E15.5 (S1, S2), and E18.5 (S1, S2). The annotations for all these six samples are generated by manually marking the anatomical regions referring to the anatomical structure in the original study (see Supplementary Fig. 1).
Spatial ATAC-RNA-seq dataset [9]. The spatial multi-omics data of mouse brain profiled by Spatial ATAC-RNA-seq platform is available at UCSC Cell Browser (https://brain-spatial-omics.cells.ucsc.edu) and GEO accession no. GSE205055. We used two samples of mouse postnatal day 21/22 (P21/P22) brains. Using the P56 mouse brain coronal section from Allen Mouse Brain Atlas ([5]) as reference, we manually annotated brain regions in P21 and utilized the annotation of P22 from [10] (see Supplementary Fig. 1).
Human lymph node dataset [7]. This dataset provides spatial ADT and RNA data using CytAssist Visium platform (10x Genomics) from SpatialGLUE [7], which is available at zenodo (https://zenodo.org/records/7879713) and GEO accession no. GSE263617.
Spatial ATAC-seq dataset [6]. The chromatin accessibility data of mice at different developmental stages were measured through Spatial ATAC-seq. This dataset can be obtained at GEO accession no. GSE214991. To achieve the integration of real non-paired data, we used the E15-rep1 sample from this dataset.

Supplementary Note 2: Implementation details

SIVA was implemented using the PyTorch library. We used the following architecture for SIVA: The default layer sizes of RNA encoder are set to (128, 64) and (64, 128) for the decoder. For the VAE used for ATAC modality, default layer sizes of (1,024, 128) were set for the encoder, and (128, 1,024) for the decoder. The latent embedding dimension was set to 20 (4 for GP and 16 for normal Gaussian) and all hidden layers were activated by ELU (exponential linear unit) function and the batch normalization technique.

We used the AdamW optimizer with a initial learning rate of 0.001 and a batch size of 512 for training SIVA. The maximum number of training epochs was set to 1000 epochs and an early stopping controller was used with a patience of 50 iterations based on the validating ELBO value. For the anchors identified by the `FindIntegrationAnchors` function, we selected those with a confidence score greater than 0.5 as the final input set. To stabilize training, each mini-batch was constructed by proportionally sampling anchor-paired spots and randomly selected spots, with a default anchor ratio of 0.3. We used the dynamic VAE technique to dynamically adjust the value of β, we set desired KL value as $0.025\times$ dimension of embedding, the minimum value of β as 4, and the maximum value of β as 25. We set the weights of MMD loss $\alpha = 5.0$ and MAG loss $\gamma = 1.0$.

Supplementary Note 3: Evaluation metrics

To comprehensively evaluate the performance of SIVA, we employed two categories of metrics: **Biological Conservation** (assessing the retention of biological heterogeneity) and **Omics Mixing** (assessing the removal of batch effects and modality integration).

Biological Conservation Metrics

Adjusted Rand Index (ARI). The Adjusted Rand Index measures the concordance between the clustering results (derived from the integrated embedding) and the ground-truth annotations, corrected for chance. An ARI of 1 indicates perfect agreement, while 0 indicates random clustering.

$$\mathrm{ARI} = \frac{\sum_{ij}\binom{n_{ij}}{2} - \left[\sum_i \binom{a_i}{2}\sum_j \binom{b_j}{2}\right]/\binom{N}{2}}{\frac{1}{2}\left[\sum_i \binom{a_i}{2} + \sum_j \binom{b_j}{2}\right] - \left[\sum_i \binom{a_i}{2}\sum_j \binom{b_j}{2}\right]/\binom{N}{2}} \tag{1}$$

where N is the total number of spots, n_{ij} is the number of spots shared between cluster i and ground-truth label j, and a_i and b_j represent the sums of the rows and columns of the contingency table, respectively.
Normalized Mutual Information (NMI). NMI quantifies the mutual information between the predicted clusters and the ground-truth labels, normalized to the range [0, 1]. It evaluates the purity of the clusters.

$$\mathrm{NMI}(U,V) = \frac{2I(U;V)}{H(U)+H(V)} \tag{2}$$

where $H(U)$ and $H(V)$ denote the entropy of the cluster assignments and ground-truth labels, respectively, and $I(U;V)$ represents the mutual information between them, defined as:

$$I(U;V) = \sum_i \sum_j \frac{n_{ij}}{N} \log \frac{N \cdot n_{ij}}{a_i b_j} \tag{3}$$

Cell Type Average Silhouette Width (cASW). cASW assesses the compactness of cell types in the latent space [8]. The final cASW score is the weighted average of $s(i)$ across all cell types, scaled to [0, 1]. A higher score indicates that cell types are well-separated:

$$\mathrm{cASW} = \frac{1}{2}(\frac{1}{N}\sum_{i=1}^{N} s(i) + 1) \tag{4}$$

Mean Average Precision (MAP). MAP evaluates the consistency of the local neighborhood for each cell type. For a given spot, it calculates the Average Precision (AP) of retrieving neighbors belonging to the same cell type in the latent space [3].

$$\mathrm{MAP} = \frac{1}{N}\sum_{i=1}^{N} \mathrm{AP}^{(i)} \tag{5}$$

$$\mathrm{AP}^{(i)} = \begin{cases} \dfrac{\sum_{k=1}^{K} \frac{\sum_{j=1}^{k} 1_{y^{(i)}=y_j^{(i)}}}{k} 1_{y^{(i)}=y_k^{(i)}}}{\sum_{k=1}^{K} 1_{y^{(i)}=y_k^{(i)}}}, & \text{if } \sum_{k=1}^{K} 1_{y^{(i)}=y_k^{(i)}} > 0 \\ 0, & \text{otherwise} \end{cases} \tag{6}$$

Graph Local Inverse Simpson's Index of Cell Types (cLISI). cLISI measures the effective number of cell types in the local neighborhood of a spot using the inverse Simpson's index. For biological conservation, we expect the local neighborhood to be pure (containing only one cell type).

$$\mathrm{cLISI}(i) = \frac{1}{\sum_{c \in C} p_{i,c}^2} \tag{7}$$

where $p_{i,c}$ is the probability of neighbor spots belonging to cell type c. The score is typically inverted or normalized such that a value close to 1 (indicating a single cell type) yields a high conservation score.

Omics Mixing Metrics

These metrics evaluate the extent to which spots from different modalities or slices are mixed, indicating the successful removal of batch effects.

Omics Average Silhouette Width (oASW). Similar to cASW, but calculated using "modality" or "batch" labels instead of cell types. Ideally, spots from different modalities should be well-mixed. To interpret this as "higher is better," we calculate:

$$\mathrm{oASW} = 1 - \left| \frac{1}{N}\sum_{i=1}^{N} s_{\mathrm{batch}}(i) \right| \tag{8}$$

A value closer to 1 indicates perfect mixing of modalities.

k-nearest Neighbor Batch Effect Test (kBET). kBET assesses whether the batch distribution in the local neighborhood of a spot matches the global batch distribution using a χ^2-test [2].

$$\chi_i^2 = \sum_{j=1}^{B} \frac{(N_{ij} - E_{ij})^2}{E_{ij}} \tag{9}$$

where N_{ij} is the observed number of neighbors from batch j, and E_{ij} is the expected number. The final metric is reported as the acceptance rate. A high acceptance rate indicates that the local batch structure is indistinguishable from the global structure.

Graph Connectivity (GC). GC quantifies whether spots of the same cell type are connected across different batches in the k-nearest neighbor (kNN) graph. It is defined as the average ratio of the largest connected component (LCC) size to the total number of spots for each cell type:

$$\text{GC} = \frac{1}{|C|} \sum_{c \in C} \frac{|\text{LCC}(G_c)|}{|V_c|} \tag{10}$$

where $|V_c|$ is the total number of spots for cell type c. A GC score of 1 implies that all spots of a given cell type are connected.

Seurat Alignment Score (SAS). This metric was used to evaluate the extent of mixing among omics layers and was computed as described in the original paper [1].

$$\text{SAS} = 1 - \frac{\bar{x} - \frac{K}{N}}{K - \frac{K}{N}} \tag{11}$$

$\bar{x}$ is the average number of same-modality neighbors among the K-nearest neighbors (computed on subsampled datasets of equal size), and N is the number of layers. With K set to 1% of the subsampled population, the score ranges from 0 to 1, where higher scores reflect better integration mixing.

Integration LISI of Omics (iLISI). iLISI measures the effective number of batches (or modalities) in the local neighborhood of a spot. Unlike cLISI, for integration purposes, we desire a high diversity of batches in the neighborhood.

$$\text{iLISI}(i) = \frac{1}{\sum_{m \in M} p_{i,m}^2} \tag{12}$$

where $p_{i,m}$ is the probability of neighbors belonging to modality m. A value close to the total number of modalities indicates excellent mixing.

Spot-level Metrics

FOSCTTM. FOSCTTM is used to assess the cell/spot-level alignment accuracy of datasets with ground-truth pairing. When the dataset contains N cells that are ordered according to their true pairings between two modalities, FOSCTTM is defined as:

$$\text{FOSCTTM} = \frac{1}{2N} \left(\sum_{i=1}^{N} \frac{n_1^i}{N} + \sum_{i=1}^{N} \frac{n_2^i}{N} \right) \tag{13}$$

Where $n_1^i = |\{j \mid d(x_i, y_j) < d(x_i, y_i)\}|$, and d is a distance metric in the integrated space.

Supplementary References

1. Butler, A., Hoffman, P., Smibert, P., Papalexi, E., Satija, R.: Integrating single-cell transcriptomic data across different conditions, technologies, and species. Nature biotechnology **36**(5), 411–420 (2018)
2. Büttner, M., Miao, Z., Wolf, F.A., Teichmann, S.A., Theis, F.J.: A test metric for assessing single-cell rna-seq batch correction. Nature methods **16**(1), 43–49 (2019)
3. Cao, Z.J., Gao, G.: Multi-omics single-cell data integration and regulatory inference with graph-linked embedding **40**(10), 1458–1466 (2022)
4. Jiang, F., Zhou, X., Qian, Y., Zhu, M., Wang, L., Li, Z., Shen, Q., Wang, M., Qu, F., Cui, G., et al.: Simultaneous profiling of spatial gene expression and chromatin accessibility during mouse brain development. Nature Methods **20**(7), 1048–1057 (2023)
5. Lein, E.S., Hawrylycz, M.J., Ao, N., Ayres, M., Bensinger, A., Bernard, A., Boe, A.F., Boguski, M.S., Brockway, K.S., Byrnes, E.J., et al.: Genome-wide atlas of gene expression in the adult mouse brain. Nature **445**(7124), 168–176 (2007)
6. Llorens-Bobadilla, E., Zamboni, M., Marklund, M., Bhalla, N., Chen, X., Hartman, J., Frisen, J., Ståhl, P.L.: Solid-phase capture and profiling of open chromatin by spatial atac. Nature biotechnology **41**(8), 1085–1088 (2023)
7. Long, Y., Ang, K.S., Sethi, R., Liao, S., Heng, Y., van Olst, L., Ye, S., Zhong, C., Xu, H., Zhang, D., et al.: Deciphering spatial domains from spatial multi-omics with spatialglue. Nature Methods **21**(9), 1658–1667 (2024)
8. Luecken, M.D., Büttner, M., Chaichoompu, K., Danese, A., Interlandi, M., Müller, M.F., Strobl, D.C., Zappia, L., Dugas, M., Colomé-Tatché, M., et al.: Benchmarking atlas-level data integration in single-cell genomics. Nature methods **19**(1), 41–50 (2022)
9. Zhang, D., Deng, Y., Kukanja, P., Agirre, E., Bartosovic, M., Dong, M., Ma, C., Ma, S., Su, G., Bao, S., et al.: Spatial epigenome–transcriptome co-profiling of mammalian tissues. Nature **616**(7955), 113–122 (2023)
10. Zhou, Y., Xiao, X., Dong, L., Tang, C., Xiao, G., Xu, L.: Cooperative integration of spatially resolved multi-omics data with cosmos. Nature communications **16**(1), 27 (2025)
